# Breastfeeding knowledge, attitudes, beliefs and practices of refugee, migrant and asylum seeker women in Portugal

**DOI:** 10.1186/s12889-024-17849-8

**Published:** 2024-02-06

**Authors:** Ana Claudia Lopes, Marisa Lousada

**Affiliations:** 1 Center for Health Technology and Services Research (CINTESIS.UA@RISE), University of Aveiro, Aveiro, Portugal; 2 School of Health Sciences (ESSUA), University of Aveiro, Aveiro, Portugal

**Keywords:** Sustainable development goals (SDGs), Breastfeeding, Complementary feeding, Refugee, Migrant

## Abstract

**Background:**

The World Health Organization recommends exclusive breastfeeding for the first six months after childbirth. However, breastfeeding is influenced by organizational, social, geopolitical, and cultural factors, which are understudied in the migrant population. This study aimed to assess the knowledge, attitudes, beliefs, and practices of refugee, migrant, and asylum-seeking mothers living in Lisbon.

**Methods:**

A sociodemographic questionnaire and a Breastfeeding Knowledge, Attitudes, and Beliefs, and Practices questionnaire were used to gather information regarding baseline breastfeeding knowledge, attitudes and beliefs, and practices towards breastfeeding.

**Results:**

Only 40% of the mothers received antenatal counselling regarding the benefits and management of breastfeeding. Of the 20 responses, 10 (50%) mothers were found to have fair breastfeeding knowledge, 14 (70%) had fair attitudes and beliefs, and 12 (60%) had fair breastfeeding practices. Correlation analysis indicated a positive correlation between mothers’ breastfeeding attitudes (*r* = 0.531, *p* < 0.05) and their breastfeeding knowledge. There was no statistically significant correlation between the mothers’ breastfeeding attitudes, beliefs, and practices.

**Conclusions:**

The findings of this study suggest that healthy breastfeeding behaviours can be stimulated by receiving proper counselling from health professionals. Countries must focus on improving breastfeeding practices, as they still fail to do all they can to promote, protect, and support breastfeeding globally. Universal interventions are necessary to improve breastfeeding, regardless of migrant or refugee status.

**Supplementary Information:**

The online version contains supplementary material available at 10.1186/s12889-024-17849-8.

## Background

The United Nations emphasizes breastfeeding’s critical role in achieving multiple Sustainable Development Goals (SDGs), improving nutrition (SDG 2) and preventing child mortality, thereby reducing the risk of noncommunicable diseases (SDG 3) [1].

The World Health Organization (WHO) recommends continued breastfeeding starting within the first hour of birth and up to two years or more to enhance child nutrition. However, globally, only approximately 44% of infants up to six months were exclusively breastfed from 2015 to 2020. From 2013 to 2018, 43% of newborns initiated breastfeeding within one hour of birth. Although 70% of women breastfeed their infant for at least one year, breastfeeding rates decline to 45% by two years of age [2].

Efforts toward meeting the target rates of breastfeeding must be increased [3] as optimal breastfeeding could save the lives of over 800,000 children annually under five years, as it prevents direct or indirect deaths through malnutrition, and potentially generates $300 billion in economic gains over 10 years [2].

Based on UNICEF program data for 82 countries, 56% of the countries have incorporated Infant and Young Child Feeding (IYCF) counselling into at least three-quarters of their primary healthcare facilities. This indicator falls below 80% of the global target by 2030 [4]. Providing counselling on IYCF by trained health professionals increases women’s knowledge and confidence in pursuing breastfeeding while allowing them to make informed decisions.

In the WHO European Region, women make up over 50% of the migrant population [5]. However, studies on breastfeeding among migrants and refugees are scarce as the implementation of projects in these settings and with these populations encompasses additional challenges. Disparities in breastfeeding rates have been reported between migrant and nonmigrant women, often varying by country of origin, the extent of stay in the new country, and acculturation [6, 7]. Addressing the health disparities of mothers and their children among those with refugee and asylum seeker status becomes challenging if these women are not reached and provided with optimal prenatal care [5].

In this study, the authors have opted to use the terms “refugee”, “migrant” and “asylum seeker” to refer to the population here studied, despite its diversity and heterogeneity, while being aware that these designations might or might not accurately reflect how people view themselves [8]. Due to the scarce studies on breastfeeding practices, attitudes, beliefs, and knowledge among refugee, migrant, and asylum-seeking mothers residing in Portugal, this research aims to evaluate these factors among such mothers living in Lisbon.

## Methods

Utilizing a quantitative research design, this study investigated breastfeeding knowledge, attitudes, beliefs, and practices among mothers with refugee, migrant, and asylum seeker status in Portugal. A structured and validated questionnaire was utilized to collect quantitative data, ensuring a systematic and comprehensive examination of the targeted variables.

Rigorous adherence to ethical standards was maintained throughout the study, aligning with established guidelines and regulations governing research practices. Namely, before the commencement of any study-related activities, ethical approval was obtained from an Ethics committee, affirming the study’s commitment to upholding ethical principles and safeguarding the well-being and rights of all participants involved. Furthermore, the fundamental ethical practice of obtaining written informed consent was diligently carried out, ensuring that all participants were provided with detailed information about the study’s objectives, procedures, potential risks, and benefits before voluntarily expressing their consent to participate.

This quantitative pilot study was conducted on mothers receiving support from a local non-governmental organization (NGO) in Lisbon between February and April 2022.

The local NGO where this pilot study was conducted is based in the capital city of Portugal, Lisbon. The Health & Wellbeing Program of that NGO exists to help migrants and refugees understand and navigate the Portuguese public health system and provide support to all members, regardless of their needs. Specifically, they provide support to all pregnant women and those who breastfeed, as they are part of a very fragile community. For recruitment, the person responsible for the Health & Wellbeing Program personally contacted the eligible women belonging to that community and invited them to appear at the NGO headquarters. One of the authors of the study was at the location and recruited all women. A total of 22 women appeared and 20 agreed to participate in the study.

The target study population consisted of women aged 18 years or older, refugees, asylum seekers, and migrants, regardless of nationality, who had at least one child at the time of the study and good proficiency in the English language. Exclusion criteria comprised women of Portuguese nationality, who were underaged at the time of the questionnaire administration, who did not have any children or who were not proficient in English.

For this study, a sociodemographic questionnaire was used that included information such as maternal age, mother’s country of origin, maternal education, and previous health conditions.

Given the absence of validated questionnaires that met the objectives and population of this study, the questionnaire used in the present study was adapted from similar studies [9–11]. Six experts (Speech and Language Therapists (SLT) with proven knowledge of infant and young child feeding) reviewed the questionnaire. Each expert panel member completed two questionnaires. The purpose of the first questionnaire was to gather data regarding their profiles. In selecting SLTs as members of the expert panel, careful consideration was given to the specialized knowledge and expertise these professionals possess in the domain of infant and young child feeding. SLTs are uniquely trained to understand the intricacies of oral-motor development and feeding behaviors. Their expertise encompasses a holistic understanding of the physiological and behavioral aspects of feeding, making them well-suited evaluators for a questionnaire focused on breastfeeding knowledge, attitudes, beliefs, and practices.

The second questionnaire was based on a set of specific questions to gather an overall evaluation of the questionnaire that was used in this study (“Mother´s Breastfeeding Knowledge, Attitudes, and Practices” questionnaire). The data were analysed using the content validity index. The average global index was 0.83, indicating excellent content validity [12].

The resulting self-administered questionnaire contained 34 questions and was divided into three subscales that addressed the participants’ breastfeeding knowledge (15 questions), attitudes and beliefs (11 questions), and practices (8 questions).

All questions were based on the participants’ experiences with their last child unless otherwise specified. A scaled scoring system was developed to categorize all studied aspects as good, fair, or poor. Each correct or favourable answer chosen by participants was given one point, and wrong/do not know/neutral answers were given zero points, which were then summed, resulting in the following scoring system:

Knowledge: Good (11–15 points), Fair (5–10 points), Poor (0–4 points).

Attitudes and Beliefs: Good (8–11 points), Fair (4–7 points), Poor (0–3 points).

Practice: Good (6–8 points), Fair (3–5 points), Poor (0–2 points).

Questionnaires were printed and given to women who met the inclusion criteria and agreed to participate in the study.

The obtained data were organized using the Statistical Package for Social Sciences (SPSS), coded, and analysed using means and standard deviation (SD) for numerical data, whereas percentages were used for categorical data. For the correlation analyses, a Pearson coefficient of 0.7. was considered acceptable, and a p-value less than 0.05 was selected as the cut-off for statistical significance.

## Results

Participants, with a median age of 33.5 [32.5,36.0] years and a significant proportion holding university or higher educational degrees (40%), predominantly comprised migrants or refugees (75%). The remaining 15% were identified as asylum seekers, primarily originating from Sub-Saharan Africa and Western and Southern Asia (85%) (Table 1).

**Table 1:** Participants’ age, birthplace, education level and status (*n* = 20)

n	Age	Birth Place	Education level*	Status
1	33	Bangladesh	Level 6	Asylum Seeker
2	33	Bangladesh	Level 6	Migrant
3	43	Pakistan	Level 4	Residency
4	30	South-Africa	Level 6	Migrant
5	34	India	Level 5	Migrant
6	30	Bangladesh	Level 7	Asylum Seeker
7	65	Siria	Level 1	Refugee
8	36	Sudan	Level 5	Asylum Seeker
9	33	India	Level 6	Migrant
10	36	Pakistan	Level 5	Migrant
11	33	Siria	Level 1	Refugee
12	30	Nigeria	Level 4	Migrant
13	36	Marrocos	Level 2	Residency
14	31	Siria	Level 3	Refugee
15	35	Ukraine	Level 6	Refugee
16	26	Ukraine	Level 6	Refugee
17	34	Afghanistan	Level 5	Migrant
18	37	Sudan	Level 4	Refugee
19	33	Gana	Level 6	Migrant
20	37	Nepal	Level 4	Migrant

*As per the European Qualifications Framework (EQF) [13]

Notably, none of the participants were currently employed, shedding light on the economic constraints within this vulnerable population. The absence of financial stability may impact healthcare-seeking behaviors and access to educational resources, including antenatal counselling on breastfeeding.

The participants were non-Portuguese speakers and encountered challenges in navigating the Portuguese healthcare system, affecting their access to maternity services and breastfeeding support.

The women’s children had a mean gestation period of 38,4 (± 2,2) weeks, and only three pregnancies (15%) were not supervised. Most children were born in their country of origin (50%) or Portugal (35%). Only one child was born in a refugee camp. The mean weight at birth was 3329 gr (± 688).

Regarding the breastfeeding knowledge of participants, a total of 10 (50%) mothers were found to have good breastfeeding knowledge, and 10 (50%) had fair knowledge.

The items that had a lower percentage of answers that align with established breastfeeding guidelines or recommendations (50%) were related to the production of milk, where only 50% (*n* = 10) said that it is common for women to produce enough milk to nourish their children; also, 70% (*n* = 14) of women said that breastfeeding is only beneficial for the child and 45% (*n* = 9) did not know that breastfeeding was beneficial for the development of orofacial structures. Finally, 60% (*n* = 12) of the mothers did not know or think that if the woman was sick, she had to stop breastfeeding her child (Table 2).

**Table 2:** Breastfeeding Knowledge of Participants (*n* = 20)

Questions	Correct answer		Incorrect answer		“Do not know” answer	
	n	%	n	%	n	%
If the mother´s breasts are small, she may not have enough milk to feed her child	18	90	1	5	1	5
It is common for women to not produce enough milk to nourish their children	10	50	8	40	2	10
Colostrum is good for child	13	65	2	10	5	25
Breastfeeding is only beneficial for the child	6	30	14	70	0	0
if the mother’s nipples are flat or inverted, she may not be able to breastfeed her child	11	55	6	30	3	15
Complementary foods should be introduced at six months of age	19	95	1	5	0	0
Breast milk is superior to formula milk in fulfilling a child’s necessary dietary requirements	18	90	2	10	0	0
Breast milk loses its benefits when it is pumped out or stored	10	50	8	40	2	10
It is good for children to be breastfed until they are 24 months of age	18	90	2	10	0	0
The food that the mother eats has no relationship to breastfeeding	12	60	8	40	0	0
Breastfeeding helps the correct development of the orofacial structures	9	45	2	10	9	45
Breast milk is sufficient for a child in the first 6 months of life	17	85	3	15	0	0
If the mother is sick, she cannot continue to breastfeed her child	8	40	7	35	5	25
Babies who are breastfed are less prone to certain diseases than children who are fed infant formula	16	80	1	5	3	15
The mother should not attempt to breastfeed her child if she is planning to return to work or study as she will not be able to have her child beside her	15	75	4	20	1	5

Delving into the breastfeeding attitudes and beliefs of participants, four (20%) mothers were found to have good breastfeeding attitudes and beliefs, 14 (70%) had fair attitudes and beliefs, and only two (10%) had poor attitudes and beliefs.

Most mothers (55%, *n* = 11) considered that breastfeeding was not hard, and 85% (*n* = 17) of them planned to breastfeed any future children. However, 60% (*n* = 12) of the participants thought that they had to stop eating certain foods because they were breastfeeding, and 60% (*n* = 12) thought they had to breastfeed even if they did not want to do so (Table 3).

**Table 3:** Breastfeeding Attitudes and Beliefs of Participants (*n* = 20)

Questions		n	(%)
Breastfeeding was/is going to be hard for me	Agree	5	(25)
	Neutral	4	(20)
	Disagree	11	(55)
I might gain weight if I breastfeed/ I did gain weight because I breastfed	Agree	4	(20)
	Neutral	6	(30)
	Disagree	10	(50)
My hair might fall because I breastfeed/ My hair fell because I breastfed	Agree	8	(40)
	Neutral	5	(25)
	Disagree	7	(35)
I do not like to give pumped breast milk to my child because it is not beneficial for him	Agree	5	(25)
	Neutral	7	(35)
	Disagree	8	(40)
I stop breastfeeding every time I take medication	Agree	2	(10)
	Neutral	2	(10)
	Disagree	16	(80)
I have to stop eating/stopped eating certain foods because I breastfed	Agree	12	(60)
	Neutral	2	(10)
	Disagree	6	(30)
I plan to breastfeed any future children	Agree	17	(85)
	Neutral	1	(5)
	Disagree	2	(10)
I think that breastfeeding classes are important	Agree	16	(80)
	Neutral	2	(10)
	Disagree	2	(10)
I think women should not breastfeed in public places	Agree	8	(40)
	Neutral	1	(5)
	Disagree	11	(55)
I think that partners might feel excluded when the mother breastfeeds	Agree	1	(5)
	Neutral	5	(25)
	Disagree	14	(70)
I think I have to breastfeed even if do not want to	Agree	12	(60)
	Neutral	4	(20)
	Disagree	4	(20)

Regarding the breastfeeding practices of participants, a total of eight (40%) mothers were found to have good breastfeeding practices, 12 (60%) had fair practices, and none of the mothers had poor practices.

Although a high percentage of mothers had breastfed their last child for any duration (75%, *n* = 15), only 40% (*n* = 8) attended or wished to attend breastfeeding classes. A remarkably high percentage (90%, *n* = 18) of women started breastfeeding within the first hour of their child’s life. However, the percentage of children who were given ready-made formulas at the hospital was also high (45%, *n* = 9). Another variable to consider is the percentage of exclusive breastfeeding up to six months (70%, *n* = 14), as well as the percentage of breastfeeding up to 24 months, which was 65% (*n* = 13) (Table 4).

**Table 4:** Breastfeeding Practices of Participants (*n* = 20)

Answers that align with established breastfeeding guidelines or recommendations	n	(%)
I attended/am attending breastfeeding classes during my pregnancy or after my delivery	8	(40)
I have previous experience with breastfeeding	15	(75)
The initiation of breastfeeding happened/ will happen immediately and within the first hour of life of my child	18	(90)
My child was not given/is not going to be given ready-made liquid formula in the hospital	11	(55)
My child was given/will be given a pacifier right after the delivery	15	(75)
I breastfed or intend to breastfeed my last child for 6 months only with breastmilk	11	(55)
I introduced or plan to introduce any foods besides breastmilk to my child before six months	14	(70)
I breastfed or intend to breastfeed my child until 24 months	13	(65)

Notably, correlation analysis revealed a positive correlation between mothers’ breastfeeding attitudes and their breastfeeding knowledge (*r* = 0.531, *p* < 0.05). Importantly, no statistically significant correlation was found between mothers’ breastfeeding attitudes, beliefs, and practices.

## Discussion

This study aimed to assess the breastfeeding knowledge, attitudes, beliefs, and practices of refugees, asylum seekers, and migrant women.

In this study, our findings shed light on the importance of promoting optimal breastfeeding practices for food security, proper nutrition, and healthy child development. We observed that only 40% of the mothers received antenatal counselling regarding the benefits and management of breastfeeding, which is in line with other studies conducted in India and South Africa [14, 15]. This highlights a significant barrier that needs to be addressed to ensure that all women, especially those from vulnerable populations, have access to comprehensive breastfeeding support. Efforts should be made to enhance the availability of breastfeeding classes and counselling services, either in the women’s home countries or in the host country, for refugees and migrant women. In Portugal, women also reported difficulties accessing breastfeeding classes, leaving this responsibility to NGOs that work with vulnerable populations. These difficulties might be related to language barriers as all women included in our study were not able to speak or read Portuguese and did not have access to official interpreters. Additionally, there is a lack of understanding about the Portuguese healthcare system, namely knowing what maternity services are available, how to access them and their rights to access the Portuguese virtually free healthcare system.

Furthermore, our study emphasizes the need to improve breastfeeding rates to enhance maternal and child health outcomes, reduce infant mortality, and prevent malnutrition. Despite the challenges faced by the participants, we found encouraging results, with a high percentage of women initiating breastfeeding within one hour of birth and exclusively breastfeeding their infants under six months compared to global averages. This suggests that cultural familiarity with breastfeeding practices among these populations may play a significant role in sustaining breastfeeding behaviours. When comparing the UNICEF data, it is possible to see that there is a high percentage (90%) of women initiating breastfeeding within one hour of birth, while the world percentage is only 43%. However, the percentage found in this study (90%) is similar to another study conducted in Portugal with Portuguese and migrant mothers where first-day in-hospital exclusive breastfeeding rates were high among both migrant and native participants (89.2% *vs*. 87.4%) but migrants were more likely to exclusively breastfeed when compared to native mothers [16].

The same is true for the percentage of infants under six months who are exclusively breastfed, where the global data indicate a percentage of only 41%, while 70% of the women in this study breastfed exclusively. In Portugal, one study with more than 100 thousand children [17] concluded that only 25% of infants under six months were exclusively breastfed. On the other hand, South Asia has one of the highest exclusive breastfeeding levels among low and middle-income regions, with Nepal, for example, reaching 64.5% of exclusively breastfed infants under six months in 2018 [18]. The same can be seen in a study conducted in Portugal with native Portuguese women and migrant women, where it was found migrant mothers had significantly higher median duration in months of any breastfeeding (Odds Ratio [OR] 6.0, 95% Confidence Interval [CI] 5.4,6.6) and exclusive breastfeeding (OR 4.0, 95% CI 3.8,4.2) than native Portuguese mothers (OR 4.0, 95% CI 3.8,4.2 and OR 3.0, 95% CI 2.9,3.0) [19]. Another possible explanation for the high rates of breastfeeding is related to maternal age in this study (35,3 (± 7,8)), as previous studies found that maternal age was a significant predictor of breastfeeding exclusivity among migrant women [20, 21].

According to other studies, ethnic minorities or women who were born outside of the countries they currently reside in are more likely to breastfeed [22, 23]. The normal demographic factors typically associated with breastfeeding seem to not matter as much as cultural familiarity with breastfeeding, which appears to be a significant determinant. The infant feeding practices of refugee mothers indicate that breastfeeding is still the preferred and typical mode of feeding in their home countries [22].

In addition, in this study, no mother was currently working, and several studies have shown that the exclusive breastfeeding status of unemployed mothers was significantly better than that of employed mothers [24, 25].

Some mothers in this study also reported that breastfeeding was needed according to tradition and religion. Certain traditional beliefs facilitate positive breastfeeding practices that persist for a longer period, especially in migrants from cultural backgrounds, in which Western breastfeeding practices were unknown or perceived as unacceptable. Studies found that mothers with stronger religious beliefs allowed them to continue breastfeeding for six months and that mothers with stronger religious beliefs had high breastfeeding initiation rates and breastfed their babies for a longer period [26–28]. In the case of the Muslim religion, the child’s right to be breastfed is affirmed by the Quaran, the source of Islamic law and morality as the Quranic verse 2:233 recommends two years of lactation [29].

In addition to considering individual cultural perceptions, the importance of migrant mothers’ knowledge emerged as a pivotal factor influencing exclusive breastfeeding. The knowledge migrant mothers possess regarding breastfeeding plays a critical role in facilitating breastfeeding duration [30–32]. This encompasses their awareness of its benefits and possessing practical knowledge to address breastfeeding challenges. Research has consistently indicated that the acknowledgement of breastfeeding benefits and the possession of relevant knowledge are linked to the actual practice of exclusive breastfeeding. Some studies have noted that mothers recognize the practical advantages of breastfeeding, viewing it as beneficial, while others perceive it as a societal expectation [33]. Contrastingly, previous investigations have highlighted certain mothers lacking an understanding of the benefits of exclusive breastfeeding [34] or possessing limited comprehension or misconceptions about the concept [35]. A deficiency in knowledge regarding breastfeeding, coupled with the disparity between ideal breastfeeding practices and real-world experiences, can lead to frustration and an increased likelihood of discontinuation [36]. It becomes evident that providing comprehensive information about the challenges associated with breastfeeding, along with emphasizing its intrinsic value, is paramount in crafting effective messages [37]. The findings in our study underscore the pivotal role of knowledge as a determining factor for the successful implementation of exclusive breastfeeding.

The correlation between mothers’ breastfeeding knowledge and their attitudes and beliefs might be related to the fact that 40% of all women in this study have higher education, as there is a universal assumption that higher maternal education leads to higher breastfeeding rates [38–40].

Concerning the percentage of women who continue to breastfeed their infants for at least one year, the numbers given by UNICEF (70%) are similar to those found in this study (75%). However, our findings also revealed areas that require attention to meet the targets of the SDGs. While the initiation and exclusive breastfeeding rates were promising, breastfeeding rates in our study (65%) declined by two years of age. This trend is consistent with global patterns (45%) and underscores the need for continued support and interventions to promote sustained breastfeeding practices beyond the first year of life.

There is also the economic aspect, where most mothers acknowledge that breastfeeding is cheaper than formula milk and choose the first option [30–32].

However, greater attention needs to be paid to the social determinants of health behaviour for this population. Lack of support is recognized as one of the largest contributors to breastfeeding challenges in refugees [22]. On the other hand, social support has been identified as having a significant positive effect on breastfeeding rates [41, 42]. The fact that the women in this study had support from a local NGO and, therefore, built over time a community of culturally matched breastfeeding peers, might have contributed to overall good breastfeeding outcomes.

One of the limitations of this study was the small sample size. Owing to the COVID-19 pandemic, obtaining permission from other institutions to recruit more participants was difficult. Therefore, results interpretation should take that into consideration. Language barriers and cultural differences may also have influenced participant responses, emphasizing the importance of cautious interpretation.

The use of self-reported data introduces the possibility of recall bias, and the reliance on a specific NGO for participant recruitment may impact the diversity of our sample. It is important to note that the authors of the study didn’t have information about the ages of the children involved. This could potentially affect the accuracy of the results. For instance, questions regarding breastfeeding habits may not necessarily reflect the actual practices but rather the intentions of the mothers to breastfeed up to 24 months.

However, this study also possesses several notable strengths that enhance the relevance of its findings. First, the research addresses a significant gap in the literature by investigating the breastfeeding knowledge, attitudes, beliefs, and practices of refugee, migrant, and asylum seeker mothers in Portugal. The inclusion of this specific population contributes valuable insights into a demographic often underrepresented in research studies, allowing for a more nuanced understanding of their unique challenges and experiences regarding breastfeeding. By focusing on this vulnerable group, the study adds depth to the existing knowledge base, fostering a more comprehensive comprehension of the factors influencing breastfeeding behaviors in diverse sociocultural contexts.

Second, the utilization of a well-structured and validated questionnaire, adapted with the input of experts in the field, strengthens the methodological rigor of the study. The thorough review and evaluation of the questionnaire by a panel of experts with expertise in infant and young child feeding enhanced the content validity of the survey instrument. This methodological precision contributes to the reliability of the data collected, ensuring that the study accurately captures the perspectives and practices of the target population.

This study’s findings hold relevance on a global scale, offering insights into the challenges faced by refugee, migrant, and asylum-seeker mothers in Portugal that may resonate with similar populations worldwide. Understanding and addressing the unique needs of these vulnerable groups is crucial across various cultural and geographical contexts [18].

The implications of our research extend to policy development, advocating for comprehensive maternal and child health policies, particularly in regions with significant migrant populations. These policies should prioritize access to antenatal counselling, healthcare resources, and culturally sensitive breastfeeding support for vulnerable populations.

Our collaboration with a local NGO underscores the effectiveness of community-based interventions in supporting breastfeeding practices. The lessons learned from this partnership emphasize the importance of community engagement, cultural sensitivity, and peer support, providing valuable insights for similar initiatives globally.

To guide the practice of healthcare providers, we recommend the integration of professional training programs emphasizing cultural competency. These programs can empower healthcare professionals to effectively communicate in diverse linguistic and cultural settings, improving the overall quality of care for migrant mothers [43].

Additionally, our findings highlight the need for knowledge dissemination through culturally sensitive channels. Developing educational materials and campaigns that resonate with diverse cultural backgrounds is crucial in promoting the value of breastfeeding [43].

We propose fostering collaborative research initiatives across borders to further explore the multifaceted factors influencing breastfeeding behaviors among migrant populations. A collective effort to generate a comprehensive evidence base will contribute to international best practices, guiding practitioners, policymakers, and researchers in diverse global settings.

## Conclusion

Healthy breastfeeding behaviours can be stimulated by receiving proper counselling from health professionals focusing on women, especially those with limited socioeconomic resources. Over the past two decades, there has been an increase in interest in infant feeding that has mostly concentrated on the sociodemographic factors related to breastfeeding, as well as the safety, cost-effectiveness, and child health outcomes. Less thought has been given to the social and cultural ramifications of such practices and the reasons behind mothers’ choice of breastfeeding or opting for artificial formula. Much of the research that does examine the complicated reasons behind breastfeeding focuses on the actions and experiences of mothers in developed countries, with little attention given to women of ethnic minorities and much less to those navigating the difficulties of poverty and refugee status. The health profiles of this population reflect their medical histories, the prevalence of diseases, and the level of medical treatment available in their country of origin or region of transit. Pregnant women require special consideration since these differences are exacerbated by pregnancy. Prenatal care and social support are essential to safe delivery and healthy mothers and babies. Thus, countries must focus on improving breastfeeding practices, as they still fail to do all they can to promote, protect, and support breastfeeding globally. Governments and international organizations are responsible for collaboratively working to support breastfeeding. However, one must recognize the importance of social and cultural factors that potentially influence breastfeeding practices among migrant and refugee women. Further investigations are needed to determine whether the observed differences are related to culturally-bound postpartum practices. Consequently, to achieve the UN SDGs related to breastfeeding and IYCF, it is paramount for governments and international organizations to collaborate in promoting, protecting, and supporting breastfeeding practices. Further research and evidence-based clinical interventions are needed to address the cultural and social factors influencing breastfeeding rates, particularly among migrant and refugee women. By prioritizing breastfeeding counseling, we can work towards achieving global breastfeeding targets and enhancing overall breastfeeding rates, contributing to the well-being and sustainable development of communities worldwide.

Moreover, future research agendas should explore the effectiveness of targeted interventions aimed at addressing the identified barriers to breastfeeding among refugees, asylum seekers, and migrant women. These interventions should consider cultural competency, language support, and the specific challenges faced by these populations. By evaluating and implementing evidence-based strategies, we can further contribute to achieving the SDGs and improving breastfeeding rates among vulnerable communities.

### Electronic supplementary material

Below is the link to the electronic supplementary material.

Supplementary Material 1

## Data Availability

The datasets used and/or analysed during the current study are available from the corresponding author upon reasonable request.
