# Supplementary material for: Breastfeeding knowledge, attitudes, beliefs and practices of refugee, migrant and asylum seeker women in Portugal
Source: BMC Public Health. 2024 Feb 6;24:394. doi: 10.1186/s12889-024-17849-8 (PMC10848452; doi:10.1186/s12889-024-17849-8)
Supplement: Supplementary file 1 — Supplementary Material 1 [file 12889_2024_17849_MOESM1_ESM.pdf]

Mother´s Breastfeeding Knowledge, Attitude, and Practices

(Lopes & Lousada, 2022)

Introduction

This questionnaire intends to gather information regarding your knowledge, attitudes, beliefs, and practices when it comes to breastfeeding. It has to be filled in by a mother of a future mother and it takes approximately ten minutes.

There are no “right” or “wrong” answers, so please, answer solely according to your opinion. Please, do not leave any questions unanswered.

I- Knowledge

1. For each of the following statements, please, mark with a cross what you think is the correct answer for you (YES, NO, or Do not know).

Statements	YES	NO	Do not know
If the mother´s breasts are small, she may not have enough milk to feed her child			
It is common for women to not produce enough milk to nourish their children			
Colostrum is good for child			
Breastfeeding is only beneficial for the child			
if the mother's nipples are flat or inverted, she may not be able to breastfeed her child			
Complementary foods should be introduced at six months of age			
Breast milk is superior to formula milk in fulfilling a child’s necessary dietary requirements			
Breast milk loses its benefits when it is pumped out or stored			
It is good for children to be breastfed until they are 24 months of age			
The food that the mother eats has no relationship to breastfeeding			
Breastfeeding helps the correct development of the orofacial structures			
Breast milk is sufficient for a child in the first 6 months of life			
If the mother is sick, she cannot continue to breastfeed her child			
Babies who are breastfed are less prone to certain diseases than children who are fed infant formula			
The mother should not attempt to breastfeed her child if she is planning to return to work or study as she will not be able to have her child beside her			

2. Where do you get your breastfeeding knowledge? (Mark with a circle one or more options)

Doctors	Nurses/midwives	Friends
Family	TV programs	Campaigns
Magazines	Breastfeeding classes	Internet
Others	If others, which?	

II- Attitudes and Beliefs

1. For each of the following statements, please, mark with a cross your opinion (agree, neutral, or disagree).

Statements	Agree	Neutral	Disagree
Breastfeeding was/is going to be hard for me			
I might gain weight if I breastfeed/ I did gain weight because I breastfed			
My hair might fall because I breastfeed/ My hair fell because I breastfed			
I do not like to give pumped breast milk to my child because it is not beneficial for him			
I stop breastfeeding every time I take medication			
I have to stop eating/stopped eating certain foods because I breastfed			
I plan to breastfeed any future children			
I think that breastfeeding classes are important			
I think women should not breastfeed in public places			
I think that partners might feel excluded when the mother breastfeeds			
I think I have to breastfeed even if do not want to			

2. If you breastfed your baby, what was(were) the reason(s)? (Mark with a circle one or more options)

Religious background	Advice from healthcare providers	Child health
Media	Cleanliness and easy preparation	Personal determination or experience
Encouragement from mother/mother-in-law	Encouragement from husband	Economic reasons
Other	If other, which?	

III – Practices

1. For each of the following statements, please, mark with a cross the answer most suitable to your last experience (YES or NO).

Statements	YES	NO
I attended/am attending breastfeeding classes during my pregnancy or after my delivery		
If not, why?		
I have previous experience with breastfeeding		
The initiation of breastfeeding happened/ will happen immediately and within the first hour of life of my child		
If not, why?		
My child was not given/is not going to be given ready-made liquid formula in the hospital		
My child was given a pacifier right after the delivery		
If yes, by whom? (mother, doctor, father, grandchild, etc)		
I breastfed or intend to breastfeed my last child for 6 months only with breastmilk		
If not, why?		
I introduced or plan to introduce any foods besides breastmilk to my child before six months		
If yes, why and which foods?		
I breastfed or intend to breastfeed my child until 24 months		
If not, why?		

Observations:
____
____
____
